# Sports Training Strategies and Interactive Control Methods Based on Neural Network Models

**DOI:** 10.1155/2022/7624578

**Published:** 2022-03-07

**Authors:** Xulei Li, Yupeng Li

**Affiliations:** ^**1**^ Department of Leisure-Sports, Pai Chai University, Daejeon 35345, Republic of Korea; ^2^College of Sports and Health, Linyi University, Linyi 276000, Shandong, China

## Abstract

Sports training strategies should be combined with science and technology to design the most suitable coaching strategies for athletes. In the current 5G Internet of Everything, the collection of wireless sensors and the deep learning of neural networks provide a new direction for the formulation of sports training strategies, guiding sports strategies to be more effective and scientific. This article aims to study and formulate sports training strategies, through the empowerment of science and technology, to better guide scientific training. With the help of the collection and sorting of sensors, the neural network allows deep learning of data, realizes human-computer interaction, and allows machines to better serve humans. This paper proposes an interactive control strategy for sports training, which enhances the interactive control of humans and machines and improves the level of training through the deep fusion of data. The experimental results of this article show that the human-computer interaction exercise training can better guide the exercise training and improve the training efficiency by 20%.

## 1. Introduction

With the continuous improvement of living standards, people are increasingly pursuing a rich spiritual civilization. Sports as a sport not only is conducive to physical exercise, improving physical fitness, but also can unite teammates and enhance team awareness. It increasingly highlights its advantages and is sought after by increasing people in the new era. However, inaccurate physical exercise will not only bring certain damage to the athlete's body, dispelling their enthusiasm for exercise, but even hinder the development of sports careers. Big data and the new era are constantly being used in life practice, and their use has pointed out the direction of scientific training for sports training. Through the deep fusion of sensors and neural networks, the machine will have better human-computer interaction, guide scientific sports training, and better help human athletes to exercise better.

In the field of sports training and interactive control, there have been research results at home and abroad. By analyzing various types of exercise and their effects on pain sensitivity, Herzig Marieherzig studied whether CT can cause exercise-induced pain reduction in young healthy men. Quantitative Sensory Test (QST) is an objective method of testing pain hypersensitivity, in which various stimuli are applied to the skin using standardized test procedures and proven equipment. The recorded response is based on the patient's response to the stimulus, thereby objectively measuring its sensitivity to the stimulus. It is not a measure of pain or sensation, but a measure of sensitivity. He selected 35 healthy men to undergo a random crossover design check before and after a single 45-minute CT and 45-minute rest time as the control conditions for passing the Quantitative Sensory Test (QST). It is concluded that a single CT has no effect on the somatosensory system of young healthy men and will not cause exercise-induced pain reduction in healthy people [1]. Zentgraf K studied the effectiveness of off-field cognitive training interventions for nonnovice, interactive sports athletes. He studied the effects of specific tasks and the transfer of effects. He analyzed the effectiveness according to the transfer level, that is, from the specific effect of the training task to the near transfer, further transfer, and far transfer intervention effects. The analysis of 16 included studies showed that about 60% of the specific training measures tested after the perception-cognitive training of sports athletes showed enhanced performance [2]. Nuhmani FIFA research investigated the influence of FIFA11+ program on sports performance parameters such as running speed, agility, and vertical jumping performance of amateur female basketball players. The study recruited and randomly selected 59 amateur female basketball players into the experimental group and the control group. The experimental group completed the 12-week FIFA 11+ program (3 times/week), while the control group members completed their regular training program. The current research shows that amateur female basketball players' sports performance parameters such as sprint speed, agility, and vertical jump performance have not improved. The lack of improvement in FIFA 11+ program performance measures indicates that the program cannot be used as a training strategy for target sports performance parameters [3]. Kai understood fighting sports from the perspective of the combination of ecological psychology and dynamic systems and studied the interactivity of fighting sports. He reviewed the experimental literature and provided preliminary support for the collaborative methods of combat sports. The literature finds that masters seem to be able to accurately perceive the boundaries of action between themselves and their opponents and the local behavior of individual combatants will lead to the synchronization of the global level of combat. However, there is still a lack of formal understanding of combat as a dynamic system, which starts with the identification sequence and control parameters. It is concluded that the ecological dynamics perspective provides a promising method to further understand the skill performance in fighting sports and assist coaches and athletes to promote the best training and learning [4]. Schulte-Frei studied pelvic floor disorders, eliminated contraindications for urinary incontinence, and outlined rehabilitation and preventive treatment methods. He conducted a systematic literature review and found that the fundamental reason for the different prevalence of incontinence lies in structure, training control, tension, hormonal status, and psychological status. These factors may not occur at the same time or even interact. It concluded that, among young female athletes, contraindicated urinary incontinence should be listed as the core issue. In addition, the training plan should include exercises and preventive measures to reduce incontinence. In addition to pelvic floor exercises, mechanical equipment may be helpful [5]. Chen X. researched and analyzed the training effects of FIFA 11+ and Harmoknee on several parameters measured by the physical performance of young amateur football players. He chose 41 young players to randomly divide each team into 2 groups. The FIFA 11+ and Harmoknee groups performed the procedure 3 times a week for 4 weeks. His control group completed their usual warm-up procedure. He evaluated 13 physical performance measures. He suggested that the traditional warm-up program of FIFA 11+ should be exchanged to male youth football players because it has a superior effect on certain neuromuscular parameters of physical performance (sprint, jumping, and stability) [6]. Bennett H compared the effects of eight-week personal exercise quality and traditional resistance training interventions on exercise quality and physical performance. He selected 46 trained adults who were randomly assigned to the quality-centered training (MQ) or traditional resistance training (TRAD) group. Personalized training is conducted twice a week for 8 weeks. The quality of exercise and physical performance were measured before and after the intervention. It concluded that although the MQ group's exercise quality score was not significantly higher, compared with traditional resistance training, exercise quality specific interventions produced comparable improvements in physical performance, but the perceived training intensity was lower [7]. The Lee HB study examined the effect of resistance training using elastic bands on the anabolic hormones and functional health of elderly women visiting the elderly welfare center in *G* City. The results showed that when they participated in the use of elastic bands, anabolic hormones, and functional fitness, there was a statistically significant difference in the interaction effects between time and groups. The results show that resistance training using elastic bands does promote the anabolic activities of elderly women and enhance their functional health [8].

This article is based on scientific sports training, and its advantages are the following: first, with the help of sensors, the data can be used to analyze the human body's kinematic function and find the law of movement. The second is to rely on the neural network model; through artificial intelligence deep learning, this article guides human-computer interaction and controls the movement training process. The third is to understand the real feelings of users, better improve experiments, guide scientific exercise, and improve the training level and ability of athletes.

## 2. Introduction to the Principles and Methods of Wireless Sensor Networks and Neural Network Models

### 2.1. Wireless Sensor Network

**Figure 1:**
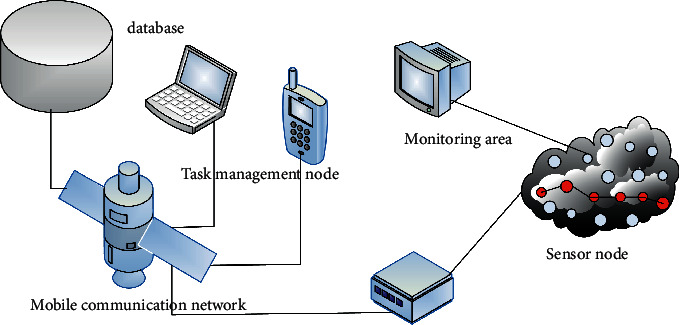
The basic structure of WSNs.

**Figure 2:**
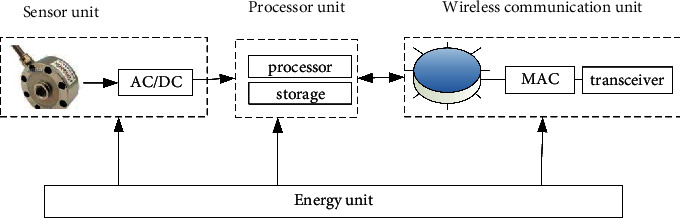
Sensor node structure.

A wireless sensor network (WSN) is a self-organizing network that detects and collects data at different nodes and then processes the data. Through the node, the sensor senses various pieces of information in the environment. With the help of the routing protocol, the information of the node is transmitted to the system terminal through the internal network, so that the relevant information of the base station can be obtained remotely, and the network operation status can be understood [9]. Its basic structure is shown in Figure 1:

The components of each node of WSN are shown in Figure 2, which are mainly composed of four parts: sensing unit (SU), data processing unit (DPU), wireless communication unit (TU), and energy unit (PU). In its composition structure, SU is responsible for collecting the required data information in the area to be tested and then using the corresponding means to send the data information to the corresponding relay site. The DPU is mainly composed of a low-power embedded microcontroller, which can coordinate various operations of the management node. For example, it processes and saves the data collected by the sensing unit. TU is mainly composed of low-power communication, etc., manages the communication process between nodes, and exchanges relevant data information at the same time. At present, PU mainly uses AA alkaline batteries or lithium batteries, which are responsible for providing energy for the work of the nodes. The energy unit is related to the lifetime of the entire network.

WSN involves many key technologies, which provide users with corresponding technical support. It mainly includes positioning technology, routing technology, clock synchronization technology, network security, and data fusion technology. These technologies have important value in the research of WSN [10].

#### 2.1.1. Positioning Technology

Node positioning technology refers to obtaining the coordinates of unknown nodes in the network through a certain positioning mechanism and then establishing the spatial relationship between nodes from the positional information. In many scenarios, only when the data information collected by a node is related to a specific location will it have a certain practical significance. Therefore, the research of many protocols in WSN is directly related to node location information and can be used to design some protocols related to node location. Some nodes are randomly distributed in the monitoring area, and their position coordinates can be located in a known way to provide the system with accurate and reliable coordinate information. Therefore, WSN can complete information acquisition and transmission through these routing protocols, thereby reducing network energy consumption.

#### 2.1.2. Routing Technology

Routing technology is also one of the key technologies in WSN. This technology can produce a better topology, and it can increase the routing efficiency of the entire network. At the same time, it can help lay a good foundation for technologies such as synchronization and positioning. More importantly, it can reduce node energy consumption and increase energy use efficiency through this technology, thereby achieving an increase in network running time. Topological structure refers to the network structure composed of network node, equipment, and communication media. The network topology defines the connection methods of various computers, printers, network devices, and other devices. The network topology includes physical topology and logical topology. The physical topology refers to the layout of various devices and transmission media on the physical structure. The physical topology usually has several types such as bus type, star type, ring type, tree type, and mesh type.

#### 2.1.3. Network Security Technology

Network security technology is the guarantee for reliable and effective reception and transmission of data information in WSN. In WSN, each node must perform fusion processing on the collected data and at the same time must complete the collaborative operation of work. Network security technology can realize the safety and reliability of data collection and processing.

Clock synchronization technology: clock synchronization technology refers to the communication protocols and applications in WSN that require the clocks of each node to be consistent. In WSN, the function of each node is limited, and the functions to be completed by the system require that each node cooperates with each other. At this time, clock synchronization technology is required to coordinate these operations. Therefore, the energy-saving clock synchronization algorithm applied to WSN is also a hot research topic underway by many researchers.

#### 2.1.4. Data Fusion Technology

In WSN, the data information between adjacent nodes has great similarity and redundancy. The forwarding of data information by each node will cause a waste of bandwidth, which correspondingly increases the energy loss of some nodes and reduces the life cycle of the network. Data fusion technology can reduce similar and redundant parts contained in the data information, to reduce the scale of the data information sent and reduce the energy consumption of the node. Therefore, the fusion technology can remove redundant information when sending data information of each node to reduce network energy consumption.

**Figure 3:**
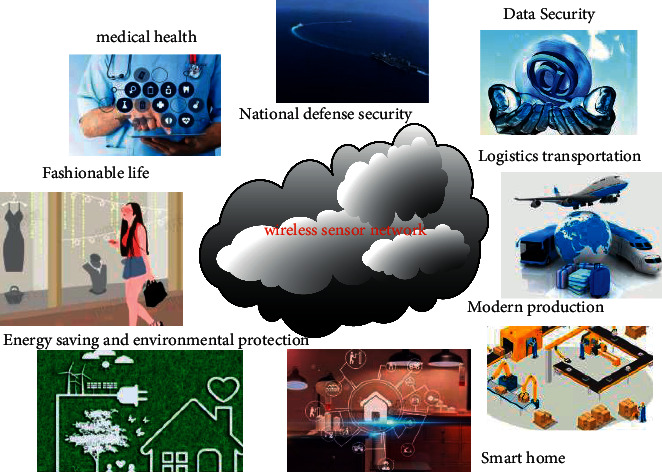
WSN application.

With the deepening of research, WSN technology has been widely used in various fields. As shown in Figure 3, the application fields of WSN are constantly expanding, and the current applications are widely used: military, medical and health, environmental monitoring, industrial, and other applications.

WSN has strong advantages and wide applications, but it also has disadvantages. WSN has yet to be studied and improved in the following aspects: (1) the battery life of the node; (2) security issues of network architecture; (3) clock synchronization in large-scale networks; (4) the ability of continuously expanding the network scale; (5) the node's data processing and transmission capabilities; and (6) miniaturization. In the future, WSN can develop towards low energy consumption, anti-interference, robustness, compatibility, low cost, and miniaturization.

### 2.2. Neural Network Model

The neural network model is essentially a directed acyclic graph model [11, 12]. The basic idea is to make the machine use the network structure model to automatically analyze a large amount of original data, extract low-level original features to achieve various combinations of high-level abstract features, and use different combinations of these features to complete specific tasks. Deep neural network refers to a neural network with a deeper level, which mainly includes three types of network structures, including fully connected neural networks, convolutional neural networks, and cyclic neural networks. At present, on the basis of neural networks, many artificial intelligence applications are designed and developed for application scenarios such as computer vision, speech recognition, and natural language processing. A revolutionary breakthrough in technology has been achieved.

**Figure 4:**
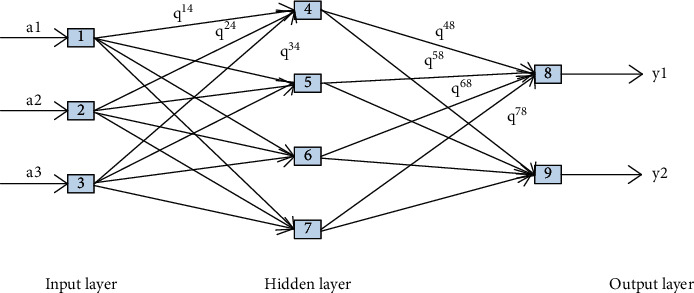
Fully connected neural network model.

The fully connected neural network model is a multilayer perceptron (MLP). The principle of the perceptron is to find the most reasonable and robust hyperplane between categories. The most representative perceptron is the SVM support vector machine algorithm. The neural network draws on both perceptrons and bionics. Generally speaking, animal nerves will send each neuron after receiving a signal, and each neuron will judge according to itself after receiving the input. After activation, the output signal is aggregated to realize the identification and classification of the information source. A typical neural network is shown in Figure 4.

Figure 4 is a typical fully connected neural network model (DNN), sometimes called a deep neural network. Different from traditional perceptrons, each node has an operational relationship with all nodes in the next layer. This is the meaning of “fully connected” in the name. The middle layer in the figure also becomes a hidden layer. A fully connected neural network usually has multiple hidden layers. Adding hidden layers can better separate the features of the data, but too many hidden layers will also increase the training time and cause overfitting.

**Figure 5:**
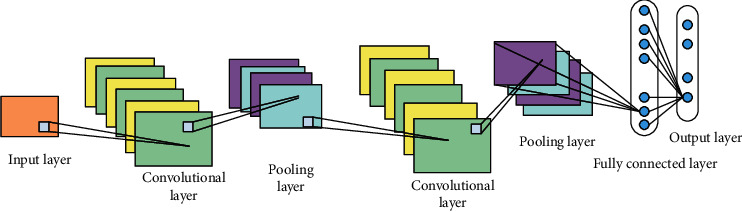
Schematic diagram of convolutional neural network structure.

Convolutional neural network is developed on the basis of traditional neural network.  Figure 5 shows a schematic diagram of a typical convolutional neural network structure. Convolutional neural network models usually include an input layer, several alternating convolutional and pooling layers, one or several fully connected layers, and an output layer. From input to output, convolutional neural network layers are connected through calculations. It transfers and extracts information layer by layer, and finally the fully connected layer classifies and outputs the classification results according to the extracted features. The main difference between CNN and traditional neural networks is that the adjacent layers are not fully connected but are locally connected through convolution operations to achieve weight sharing. This greatly reduces the number of parameters. In addition, a pooling step is added after convolution to adjust the output of this layer. The pooling operation is a special operation in the convolutional neural network, which is mainly to propose the key information of the area in a certain area. Its operation often appears after the convolutional layer, which can reduce the number of feature outputs by the convolutional layer, thereby reducing the model parameters and improving overfitting. The pooling operation is composed of two key variables: the pooling template and step size. The template describes the size of the extracted information area, which is generally a square window. The step length describes the moving step length of the window on the output feature map of the convolutional layer and is generally equal to the side length of the template.

The idea of cyclic neural network construction is to make full use of the sequential information of the data in the neural network. Calculate the data of the input layer through internal synchronization to ensure the relevance of data calculations. In life, user usage data can be abstracted as sequence data. With the help of cyclic neural networks, businesses can find users' interests and achieve accurate push. Recurrent neural network has shown great power in the task of processing sequence data and has become a very popular model for processing sequence data.

**Figure 6:**
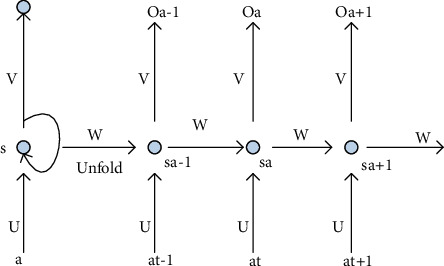
Schematic diagram of the unfolded recurrent neural network structure.

Figure 6 shows the RNN expanded into the complete network, and the RNN is constructed by expanding into complete sequence data. The training of RNN is similar to traditional neural networks. It still uses the backpropagation algorithm, referred to as BP, which is a learning algorithm suitable for multilayer neuron networks. It is based on the gradient descent method. The input-output relationship of the BP network is essentially a mapping relationship: the function of a BP neural network with *n* inputs and *m* outputs is a continuous mapping from n-dimensional Euclidean space to a finite field in m-dimensional Euclidean space. This mapping is highly nonlinear. However, there is a little difference, because the network parameters of the RNN are shared at all times. Therefore, each output depends not only on the current moment, but also on all previous moments.

The neural network includes an input layer on the far left, an output layer on the far right, and multiple hidden layers in the middle. It is composed of multiple neurons combined with multilayer perceptrons. Assuming that a neuron has three input values of *a*_1_, *a*_2_, *a*_3_ and a bias term *m*, *Q*_1_, *Q*_2_, *Q*_3_ represents the weight of each input and the offset term is the intercept of the function, which controls the distance where the function deviates from the origin. *f*(*∗*) is an activation function, and sigmoid and ReLU are commonly used. The output value *J* of the arithmetic unit is shown in the following formula:

(1) $$\begin{array}{c} J=f\left(Q^{R}a\right)=f\left(\overset{3}{\underset{x=1}{\sum }}Q_{x}a_{x}+m\right). \end{array}$$

The neural network model needs to initialize the training parameters during training and then update the parameters according to the learned features during training. A simple initialization is to uniformly and randomly select the value of each parameter from a certain interval according to the Gaussian distribution. Gaussian distribution refers to a normal distribution, also called “normal distribution.” It is a probability distribution that is essential in the fields of mathematics, physics, and engineering and has a significant influence in many aspects of statistics. If the random variable *X* obeys a normal distribution with a mathematical expectation of *µ* and a variance of *σ*2, its probability density function is a normal distribution, the expected value *µ* determines its position, and its standard deviation *σ* determines the magnitude of the distribution. When *μ* = 0 and *σ* = 1, the normal distribution is the standard normal distribution. In further related research, a better random initialization method for training is proposed. This method normalizes the weight to the interval [−*c*, *c*], and the calculation of *c* is as follows:

(2) $$\begin{array}{c} c=\sqrt{\frac{6}{G_{m}+G_{m+1}}}. \end{array}$$

Among them, *G*_*m*_ and *G*_*m*+1_ are the hidden layer size before and after the weight.

When training a deep neural network, each hidden layer should use a nonlinear activation function *f*(*x*) for data mapping. This is because the combination of multilayered linear functions essentially only has the expressive power of linear functions (for example, combining multiple linear formulas together only produces another linear formula). Therefore, when the activation function is linear, compared with a single hidden layer neural network, a deep neural network containing multiple hidden layers does not increase the expressive power. The sigmoid function is a very commonly used nonlinear activation function, the function value range is [0, 1], and its formula is as follows:

(3) $$\begin{array}{c} h\left(i\right)=\frac{1}{1+e^{-i}}. \end{array}$$

**Figure 7:**
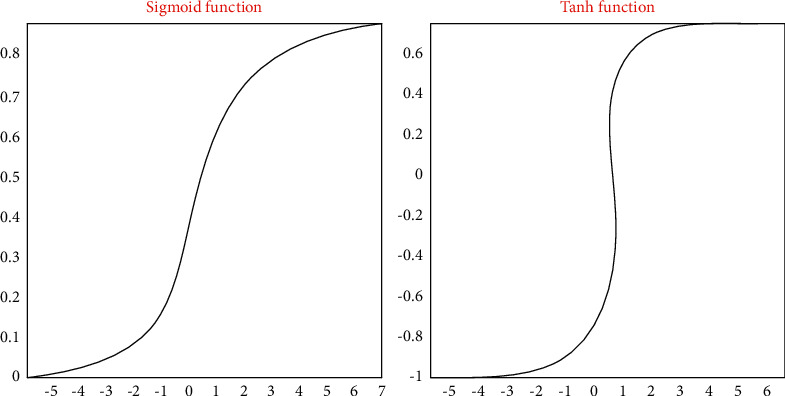
Comparison of sigmoid function and Tanh function.

The hyperbolic tangent function Tanh is a variant of the sigmoid function. The function value range is [−1,1], and its calculation is shown in formula (2) and the following formula. The graphical comparison of these two functions is shown in Figure 7.

(4) $$\begin{array}{c} \text{Tanh}\left(i\right)=\frac{e^{i}-e^{-i}}{e^{i}+e^{-i}}. \end{array}$$

The Rectified Linear Unit (ReLU) is another nonlinear activation function, and its design is similar to biological nerves. The calculation process is shown in the following formula:

(5) $$\begin{array}{c} \text{ReLU}\left(i\right)=\max\left(0,i\right). \end{array}$$

Overfitting refers to the overtraining of the model, a fitting function that fits the distribution of the training set, which reduces the accuracy of prediction for new data [13]. To solve this problem, we can consider setting the attenuation coefficient when updating the weights, so that the complexity of the network is reduced, thereby improving its generalized expression ability. T1 regularization (T1 regularization) and T2 regularization (T2 regularization) rely on adding a regularization term after the loss function to attenuate the network weight when the gradient is returned. The calculation formulas of the loss function are shown in the following equations, respectively, where *γ* represents the weight of the attenuation network, and *j* is the loss function.

(6) $$\begin{array}{c} T_{1}=T+\frac{\gamma }{m}\underset{j}{\sum }\left|j\right|, \end{array}$$

(7) $$\begin{array}{c} T_{2}=T+\frac{\gamma }{2m}\underset{j}{\sum }j^{2}. \end{array}$$

Another way to prevent overfitting is to directly modify the design of the network and randomly drop a certain percentage of hidden layer neurons during network training. This method is called Dropout [14]. The specific process is to clear the output value of the hidden layer node with a certain probability of 1 − *p* during the network training process. When the gradient is returned to update the weight, the weight connected to the node is no longer updated. The zero-clearing process is expressed as the following formula:

(8) $$\begin{array}{c} g=d\cdot k\left(Lg\right). \end{array}$$

Among them, *g* ∈ *D*^*m*×1^, *L* ∈ *D*^*e*×*m*^, *d* ∈ *D*^*m*×1^, and function *k*(0) = 0.

The loss function is used in machine learning to estimate the gap between the predicted value and the true value and is generally a nonnegative real value function. Usually, it is expressed by *S*(*A*, *h*(*i*)); the smaller the function, the better the robustness of the learned parameters. In addition to the most common 0-1 loss function, the square loss function, and absolute value loss function that directly use the difference between the predicted value and the true value to calculate, the following loss functions are also often used.

#### 2.2.1. Log-Likelihood

Since the likelihood function of independent and identically distributed samples has the form of continuous product, it is convenient to obtain the partial derivative after taking the logarithm of the likelihood function. Therefore, in practice, the equivalent form of the likelihood equation is often used, which is called the log-likelihood equation. The standard form of the loss function designed using log-likelihood is*S*(*A*, *P*(*A|B*))=−log  *P*(*A|B*), which means that the probability *P*(*A|B*) reaches the maximum value when the sample A is in the classification B. In an intuitive sense, it is to use the distribution of known samples to find the most likely parameter value to be able to predict the predicted value that conforms to this distribution [15]. For the sample set *B*={*b*_1_, ..., *b*_*m*_}, the formula can be transformed into

(9) $$\begin{array}{c} S\left(A,P\left(A|B\right)\right)=-\log\;P\left(A|b_{1},\ldots ,b_{m}\right)=-\overset{m}{\underset{x=1}{\sum }}\log\;P\left(b_{m}|A\right). \end{array}$$

The log-likelihood loss function is often used to use Softmax regression as the loss function of the model of the last layer of the network model. The Softmax logistic regression model is an extension of the logistic regression model to multiclass problems. In a multiclassification problem, the class label *y* can take more than two values. The Softmax regression model is very useful for problems such as MNIST handwritten digit classification. The purpose of this problem is to identify 10 different single digits.

#### 2.2.2. Cross-Entropy

The cross-entropy loss function can speed up the weight update speed during network model training. The calculation of the cross-entropy loss function for a neuron in the network (multiple input and single output) is shown in

(10) $$\begin{array}{c} T=-\frac{1}{m}\underset{i}{\sum }\left[b\;\ln\;c+\left(1-b\right)\ln\left(1-c\right)\right], \end{array}$$

where *b* is the true value output by the neuron and *c* is the predicted value. When the network model uses the sigmoid function as the activation function, the speed of updating the weights through the cross-entropy loss function is affected by the size of the error. The larger the error, the faster the update speed.

Gradient descent (GD) is the most common algorithm used as an optimization method for network model training. Its goal is to minimize the loss function *S*(*λ*) by updating the weight *λ* ∈ *D*^e^ [16]. The way to update the weight is to adjust the value of the weight in the positive direction according to the gradient ∇_*λ*_*S*(*λ*). The learning rate *ε* determines the step size of the weight update. The formula for updating the weights of the most general batch gradient descent is as follows:

(11) $$\begin{array}{c} \lambda =\lambda -\varepsilon •\nabla_{\lambda }S\left(\lambda \right). \end{array}$$

#### 2.2.3. Stochastic Gradient Descent (SGD)

For each training sample *b*^*a*^ and d^*a*^ labeling, the formula for updating the weight of the optimization method is as follows:

(12) $$\begin{array}{c} \lambda =\lambda -\varepsilon •\nabla_{\lambda }S\left(\lambda ,b^{a},d^{a}\right). \end{array}$$

Compared with batch gradient descent, this optimization method uses only a pair of samples and annotations to calculate the gradient of the loss function at a time, which is faster than the latter using the entire data set to calculate the gradient. However, due to the small number of calculated samples, the update effect may not be good.

#### 2.2.4. Minibatch Gradient Descent (SGD)

Minibatch gradient descent takes the best of the two methods to be combined. For each minibatch containing *w* training samples, the formula for updating the weight of this method is as follows.

(13) $$\begin{array}{c} \lambda =\lambda -\varepsilon •\nabla_{\lambda }S\left(\lambda ,b^{a:a+w},d^{a:a+w\text{)}}\right). \end{array}$$

For the three basic gradient descent methods, the initial learning rate needs to be continuously adjusted to avoid nonconvergence of model training.

#### 2.2.5. Gradient Descent Based on Momentum (GD with Momentum)

With the training of the model, the gradient of the actual function is constantly changing, making the training convergence slower and slower. Therefore, the momentum term is set to speed up the decline during iteration. Momentum is also called linear momentum. In classical mechanics, momentum is expressed as the product of the mass and velocity of an object. It is a physical quantity related to the mass and speed of an object and refers to the effect of a moving object. The process of this method to update the weight is as follows:

(14) $$\begin{array}{c} z_{t}=\delta z_{t-1}+\varepsilon •\nabla_{\lambda }S\left(\lambda \right),\\ \lambda =\lambda -z_{t}. \end{array}$$

The coefficient *z* of the momentum term is generally set to 0.9.

#### 2.2.6. Adaptive Moment Estimation (Adam)

All the methods need to manually set the learning rate or use a common method of artificially attenuating the learning rate to reduce the learning rate according to the number of training iteration steps. The optimization method is an adaptive optimization method that can adjust the learning rate according to each parameter [17]. First, for the number of iteration steps *f* and the weight item *λ*_*x*_, assume that there is a variable *f*_*t*,*x*_=∇_*λ*_*S*(*λ*_*x*_), which is represented by *f*_*t*_ for the convenience of presentation. This method is similar to the momentum method, and the attenuation term of the gradient is also set as follows:

(15) $$\begin{array}{c} p_{t}=\alpha_{1}p_{t-1}+\left(1-\alpha_{1}\right)f_{t},\\ q_{t}=\alpha_{2}q_{t-1}+\left(1-\alpha_{2}\right)f_{t}^{2}. \end{array}$$

Among them, *p*_*t*_, *q*_*t*_ estimates the mean value of the gradient and the uncentered variance, and both are initialized with a 0 vector. When the coefficient *α*_1_, *α*_2_ is set close to 1, the two items gradually deviate to 0 with training. To avoid this offset situation, we set the following calculation.

(16) $$\begin{array}{c} {\hat{p}}_{t}=\frac{p_{t}}{1-\alpha_{1}^{t}},\\ {\hat{q}}_{t}=\frac{q_{t}}{1-\alpha_{2}^{t}}. \end{array}$$

Finally, using the calculated value to update the weight, the formula is as follows:

(17) $$\begin{array}{c} \lambda_{t+1}=\lambda_{t}-\frac{\varepsilon }{\sqrt{{\hat{q}}_{t}+\kappa }}•{\hat{p}}_{t}. \end{array}$$

The default values of several coefficients *α*_1_, *α*_2_, *ε* of this method are 0.9, 0.999, and 10^−8^, respectively. Therefore, far, this optimization method has been proved to be one of the best optimization methods in the training of most deep neural network models.

### 2.3. Sports Training Strategies

Sports training is a planned and organized special activity under the guidance and education of the coach, which aims to improve the athlete's ability and performance. Sports training management is the use of different management methods to improve athletes' performance and work efficiency based on sports training in special activities. It achieves the goal of sports management, and sports training management is a comprehensive training process that is planned, controlled, organized, and coordinated [18].

**Figure 8:**
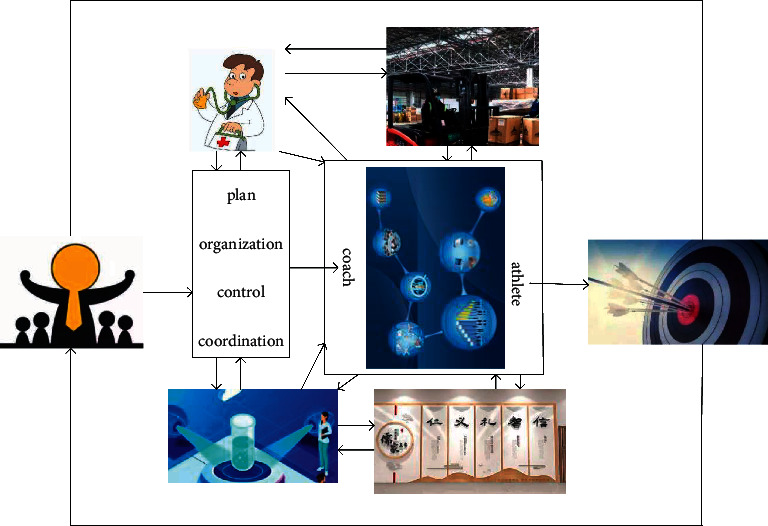
Sports training management system model.

According to the different control variables, the system can be divided into two types: natural systems and man-made systems [19]. From the definition of the sports system, we can know that the sports training management system consists of two parts: the managers and the person being managed. It is a man-made system, and the key is to improve athletes' performance and achieve established goals through scientific guidance, as shown in Figure 8.

From Figure 8, we can see that the factors that make up the sports training management system mainly include people, finance, materials, time, and information. People here are not just athletes, they include coaches, doctors, administrative staff, and scientific researchers. The money mentioned here mainly refers to the funds required for sports training, competition, venue construction and maintenance, etc. It is the basis for ensuring the normal training of athletes.

Training strategy is the targeted and purposeful training methods and implementation steps that we take to achieve the goal. It adopts a comprehensive set plan of unbounded media, organizational forms, and action measures [20]. It is divided into four training strategies: method type, content type, style type, and task type. The method type is divided into two types: lecture type and discovery type. The content type is divided into four types: linear type, branch parallel, loop type, and comprehensive type. The style is divided into three strategies: style-centered, coach-centered, and athlete-centered. The task type is divided into several types: arranging task, training strategies based on exercise content, practicing strategies, problem-oriented strategies, and explanatory strategies [21].

### 2.4. Principles of Interactive Control

Motion capture is to capture human motion information with the help of sensors and realize the transformation from information to data by analyzing and processing the information, which is used for better motion simulation and interactive control [22]. Motion capture equipment usually includes sensors, signal capture equipment, data transmission equipment, and data processing equipment. Motion capture technology has abundant application scenarios in life, such as computer animation, sports training, sports analysis, and distance education.

The motion capture system exerts a strong advantage in sports training. With the help of wide-angle and comprehensive data observation records, trainers can grasp the habits, strengths, and weaknesses of athletes and formulate scientific training programs accordingly. Athletes can also understand their own level, improve their abilities in a targeted manner, make up for deficiencies, and better improve their abilities and performance. At present, motion capture technology is widely used in sports training and has achieved solid results.

## 3. Experiment and Analysis of Sports Training Strategies and Interactive Control Methods

### 3.1. Experimental Design

This article takes 24 athletes and their coaches as the subjects of investigation to study their training methods. By consulting books and papers related to sports training strategies, this article designs sports training strategies. This article designs two targeted questionnaires for athletes and coaches. It mainly understands the basic situation of athletes, achievements, training methods, training plans and technical guidance, psychological adjustment training, diet adjustment programs, music matching, and other prematch matters to conduct investigation and research. The purpose is to better understand the training plans adopted by athletes and coaches in sports training.

### 3.2. Experimental Implementation Process

After understanding the relevant situation of the athlete, it collects the relevant data information of the athlete through the motion capture sensor as the initial data. It imports the athlete's data information into the computer, inputs various control operation training programs, and simulates the athlete's movement through the computer's deep learning, controls the relevant parameters to remain unchanged, collects relevant data, and develops the best sports design plan. After completing the program design, it asked relevant experts, scholars, and lecturers for their opinions and suggestions on the exercise program. After the modification, the athletes are requested to perform relevant training in accordance with this plan, and the data after the athletes' training is collected.

### 3.3. Data Analysis and Processing

It can be seen from Table 1 that most athletes are within 30 years of age and some are over 30. There is little difference in their height and weight. In general, the training time of the athletes is not long, and the performance of various indicators is balanced.

It can be seen from Table 2 that the arrangement strategies of athletes at different ages are different. At a young age, taking into account the physical development, it is not suitable for high-intensity training. At this time, basic training is mainly used to enhance physical fitness. As we grow older, we begin to increase the intensity of training. At this time, we focus on intensive training. At this time, athletes can choose the appropriate field to carry out targeted training according to their own advantages. In the later stage, the main focus is on targeted training. Athletes choose to strengthen their advantages and improve their abilities based on their own advantages.

It can be seen from Table 3 that the diet of athletes emphasizes more eating smaller meals and maintaining energy balance by evenly distributing them. The psychological requirement is to maintain a stable state of mind and not to be affected by foreign objects. Music training 2-3 times a week can help athletes soothe their emotions and relax themselves. Generally speaking, athletes are fairly satisfied with the various supporting technical services, but sometimes the results may be different.

**Figure 9:**
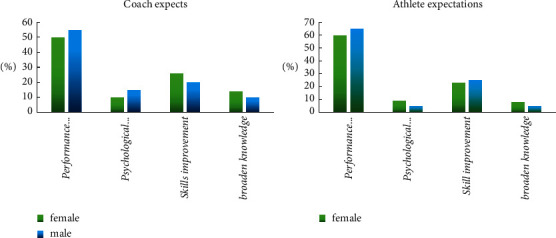
Comparison of expectation differences between trainers and athletes on training programs.

It can be seen from Figure 9 that there are certain differences in the arrangement of training strategies between trainers and athletes. For trainers, although performance is important, it is more about training mental stability, improving skills, and increasing knowledge. For athletes, they pay more attention to performance improvement, and they firmly believe that their mentality is good enough. There are also certain differences in training strategies between male and female athletes. Male athletes are more competitive and have a strong demand for performance improvement. Compared with male athletes, female athletes pay more attention to skill improvement and gaining knowledge.

**Figure 10:**
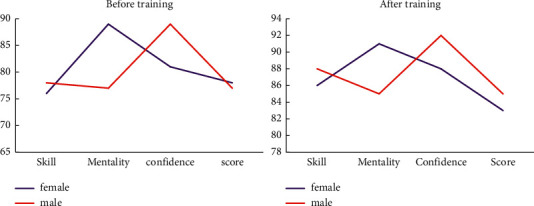
Changes of various indicators before and after the athletes' training.

It can be seen from Figure 10 that all indicators of athletes have increased after training. Particularly, for male athletes, the increase in all aspects is more obvious. Compared with male athletes, female athletes have slightly improved in all aspects, but overall they are slightly inferior. It can also be seen from the side that female athletes have a more stable mentality and a clearer understanding of their own situation.

**Figure 11:**
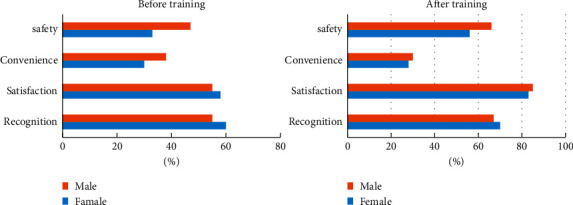
Trainer's evaluation of the motion capture analysis system.

It can be seen from Figure 11 that the overall evaluation of the sports tracking analysis system by the trainers is not high, although the impression of the sports tracking analysis system has changed after trying. However, in general, they do not approve the convenience and safety of the motion tracking analysis system. In the future, further safety tests can be used to prove the safety of the product.

**Figure 12:**
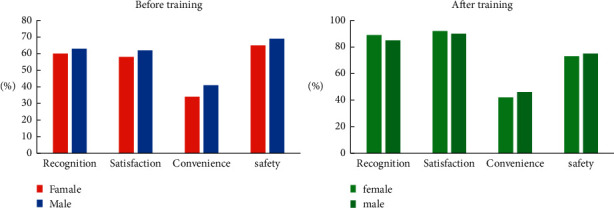
Athletes' evaluation of the motion capture analysis system.

It can be seen from Figure 12 that the overall evaluation of the sports tracking analysis system by the athletes is good. For sports young people, they are more willing to accept new things and are more likely to have a good impression of the sports tracking analysis system. However, in the same way, athletes and trainers do not recognize the convenience of the sports tracking analysis system, and they all have concerns about the security risks of the sports tracking analysis system. In the future, it can be optimized based on this improvement.

## 4. Discussion

This article carried out a test of the sports tracking analysis system on 24 athletes. After understanding the basic information of the athletes, the relevant variables were controlled to verify the performance of the sports tracking analysis system. Through experiments, we found that the sports tracking analysis system plays a great role in improving athletes' performance and skills and stabilizing their mentality. After testing, it is found that the survey trainers and athletes have a high degree of recognition and satisfaction with the sports tracking analysis system. However, they have certain concerns about the convenience and safety of the motion tracking analysis system. In addition, the motion tracking system also has the problem of excessively high prices. In the future, software and hardware can be optimized to improve the performance of the motion tracking analysis system, reduce costs, reduce weight, and increase convenience. At the same time, it adds related safety tests to ensure its safety.

## 5. Conclusion

In this paper, the exercise training strategy and interactive control methods of wireless sensors and neural network models are studied. The performance of the sports tracking analysis system is verified through experiments, and this article has found a good method suitable for athletes' training. Through investigating the psychological feelings of trainers and athletes, it also provides directions for improving the sports tracking analysis system. However, the experiment also has certain problems: too few samples of athletes are selected in the experiment, too few items are in the experiment, and there may be problems with low accuracy and low credibility of the results. The experiment is only a general one on sports, and no specific sports analysis is selected. There are too few items in the experiment, and there is a problem that the accuracy of the results is not high.

## Figures and Tables

**Table 1:** Questionnaire on the basic situation of athletes.

	Age (in years)	Weight (kg)	Height (m)	Training time (h)	Quantity (person)
Female	16–30	80–100	1.62–1.77	1–3	12
Male	16–35	100–140	1.75–1.89	1–5	12
Total	16–33	90–120	1.69–1.83	1–4	24

**Table 2:** Training plans and arrangements for athletes.

	Basic training (%)	Intensive training (%)	Targeted training (%)
16–18	60	30	10
18–25	30	35	35
25–30	25	30	45

**Table 3:** Supporting the situation of supporting services for athletes' training.

	Diet strategy	Psychological strategy	Music support
Schedule	5-6 times a week	1-2 times a month	2-3 times a week
Requirement	High protein diet	Stable mind	Relaxation
Finished condition	95%	92%	89%

## Data Availability

The data underlying the results presented in the study are available within the manuscript.
